# Caregiver Support for Asian American Alzheimer’s Disease and Related Dementias Caregivers: A Conceptual Synthesis

**DOI:** 10.1093/geroni/igab046.253

**Published:** 2021-12-17

**Authors:** Mengyao Hu, Allison Squires, Bei Wu

**Affiliations:** New York University, New York, New York, United States

## Abstract

Informal caregiver support has been defined as different types of interventions. However, it has not been well explained in the social context and not well discussed as an integrative concept for dementia caregivers who are Asian Americans. Therefore, the aim of this study was to conduct a dimensional analysis--a type of evidence synthesis--to explore caregiver support in the context of Asian American dementia caregivers. A synthesis of 40 articles produced four interrelated dimensions of caregiver support: Individual (language, information, psychological issue, and culture); Family (family member support, availability of extended family, and decision making); Community (bilingual and bicultural help, and religion and spiritual source); and Professional healthcare system (expectations from healthcare professionals and caregivers for caregiver interventions, communication concordance, initiative in seeking help, and trust). The findings provide guidance for future studies on this population in promoting caregiver’s health and developing caregiver interventions.

